# Manual Massage Therapy for Neuropathic Pain: Clinical Evidence and Mechanistic Insights

**DOI:** 10.1155/prm/4403173

**Published:** 2026-06-27

**Authors:** Yiting Guo, Yi Zhong, Xin Yun Chia, Xiaoqiu Wang, Bin Xiao

**Affiliations:** ^1^ School of Acupuncture–Moxibustion and Tuina, Shanghai University of Traditional Chinese Medicine, Shanghai, China, shutcm.edu.cn

**Keywords:** analgesic mechanisms, brain functional remodeling, inflammatory response, ion channels, massage, neuropathic pain (NP), synaptic plasticity

## Abstract

Neuropathic pain (NP) is a complex chronic pain condition arising from a lesion or disease of the somatosensory nervous system, presenting significant clinical challenges with limited therapeutic efficacy from conventional pharmacological interventions. Massage, as a specific form of manual therapy, is a pivotal therapeutic modality in traditional Chinese medicine (TCM) and has been increasingly adopted for the management of NP due to its noninvasive nature and favorable safety profile. Recent advances in clinical and basic research indicate that massage not only effectively mitigates pain symptoms and ameliorates functional impairment and emotional disturbances but also exerts analgesic effects by modulating inflammatory responses, regulating ion channel function, controlling synaptic plasticity at the spinal level, and inducing brain functional remodeling. This review synthesizes and critically evaluates current evidence regarding the application of massage for NP, integrating findings from both animal studies and clinical trials. It systematically analyzes potential molecular and cellular mechanisms, including the HMGB1/NF‐κB pathway, mechanosensitive Piezo channels, the astrocytic NDRG2/GLT‐1 pathway, and the default mode network (DMN). By critically synthesizing the underlying mechanisms of massage, this review aims to provide a theoretical basis for clinical promotion and mechanistic research, thereby advancing the development of comprehensive, potentially disease‐modifying therapeutic strategies for NP.

## 1. Introduction

Neuropathic pain (NP) is a complex chronic pain state and has long remained a significant challenge in pain medicine worldwide. According to the latest definition from the International Association for the Study of Pain, NP is explicitly defined as “pain caused by a lesion or disease of the somatosensory nervous system” [1, 2]. This definition highlights a fundamental distinction from nociceptive pain. It underscores that NP is not simply a secondary manifestation of tissue injury but instead arises from structural or functional pathological alterations within the somatosensory system. Such alterations can drive maladaptive neuroplasticity and contribute to persistent pain even after the initial insult has resolved [3, 4].

From an epidemiological perspective, the burden of NP continues to increase. Global epidemiological surveys suggest a prevalence of approximately 7%–10% in the general population [5]. However, the prevalence is substantially higher in specific high‐risk groups. For example, about 20%–50% of patients with diabetes may develop painful diabetic peripheral neuropathy (DPN) [6, 7]; in patients with herpes zoster, the incidence of postherpetic neuralgia (PHN) also remains high. Recent surveys report that the prevalence of NP in the general population is 3.0%–17.0% in China [8], 7%–8% in Europe [9], and approximately 10% among adults in the United States [10]. Notably, variations in case definitions, sampling strategies, and diagnostic screening tools may partly explain the wide range reported across regions and studies. Moreover, NP frequently coexists with anxiety, depression, sleep disorders, and cognitive decline, creating a vicious cycle that severely impairs quality of life and social functioning and imposes a substantial economic and societal burden [3, 11].

Currently, international authoritative guidelines (e.g., the NeuPSIG guidelines) recommend first‐line pharmacotherapies that primarily include tricyclic antidepressants (TCAs), serotonin–norepinephrine reuptake inhibitors (SNRIs), and calcium channel modulators (gabapentin and pregabalin) [12]. Nevertheless, clinical efficacy is often modest, and adverse effects are common. Systematic reviews indicate that the number needed to treat (NNT; the number of patients who must be treated for one to achieve ≥ 50% pain relief) for these first‐line agents typically ranges from 3.6 to 7.7 [13]. This suggests that a considerable proportion of patients still fail to achieve adequate analgesia with a single agent and may require dose escalation, switching, or combination strategies in clinical practice. More importantly, these agents are associated with notable side effects. For instance, TCAs may cause dry mouth, constipation, and cardiotoxicity; pregabalin and gabapentin frequently induce somnolence, dizziness, peripheral edema, and cognitive blunting [12]. Although opioids have strong analgesic potency, they carry serious risks such as tolerance, addiction, and opioid‐induced hyperalgesia, resulting in an unfavorable risk‐benefit profile for long‐term use [14]. Collectively, these limitations highlight the need for complementary, potentially disease‐modifying nonpharmacological strategies that provide analgesia while minimizing systemic adverse effects.

Massage is a widely practiced therapeutic modality and represents a specific form of manual therapy, with diverse cultural expressions across regions. Its nomenclature reflects local traditions: It is commonly referred to as Swedish massage in Europe, Thai traditional massage in Southeast Asia, and practices such as Shiatsu, Anma, or acupressure in Japan. In China, massage, also known as Tuina, it represents a longstanding therapeutic approach rooted in traditional Chinese medicine (TCM), where it has been systematically developed into a structured form of manual therapy. This approach integrates a range of techniques targeting soft tissues, joints, and specific acupuncture points and has historically been applied to the management of musculoskeletal disorders, neurological conditions, and pain‐related syndromes. In the Chinese medical context, massage is not merely a wellness practice but a regulated clinical intervention, typically administered by trained practitioners with appropriate medical qualifications or prescription authority. In contemporary clinical settings, massage shares fundamental biomechanical principles with other forms of manual therapy, including spinal manipulation and soft‐tissue mobilization. These interventions are characterized by the application of controlled mechanical forces to biological tissues, thereby influencing neuromuscular function, circulation, and neurophysiological processes. At the same time, massage retains distinctive features through its integration with TCM theoretical frameworks, particularly meridian theory and acupoint‐based manipulation. These conceptual foundations provide a structured rationale for the selection of treatment sites and techniques, thereby distinguishing massage as a specific modality within the broader framework of manual therapy [15]. With advances in mechanobiology, the therapeutic effects of manual mechanical interventions, including massage, are increasingly understood through the framework of mechanotransduction, whereby external mechanical forces are translated into intracellular biochemical signals that regulate cellular function and tissue homeostasis. Accumulating evidence indicates that such mechanical stimulation can modulate pain‐related processes at multiple levels, including the regulation of mechanosensitive ion channels involved in neuronal excitability (e.g., Piezo family proteins) [16], improvement of local microcirculation and neurotrophic factor expression [17], and modulation of spinal and supraspinal pain‐related neural networks via somatosensory inputs [18]. Together, these findings support the concept that massage functions as a biomechanical signal input capable of influencing pain across peripheral, spinal, and supraspinal levels.

A key advantage of massage in NP is its multitarget, holistic regulatory properties. Rather than blocking a single pain pathway, massage exerts comprehensive analgesic effects across multiple levels, including improving the local microenvironment, modulating spinal signal transduction, and reshaping functional brain networks. As a nonpharmacological and noninvasive approach, massage may have the potential to influence disease progression while avoiding medication‐related adverse effects. Most existing reviews focus on single mechanisms or clinical applications; this review aims to integrate and critically discuss the multilevel regulatory cascade of “peripheral inflammation—ion channels—spinal plasticity—brain functional remodeling” and to link these mechanisms to translational bottlenecks, thereby bridging basic research and clinical practice. This article is presented as a narrative review aimed at synthesizing current clinical and mechanistic evidence on massage therapy for NP.

### 1.1. Literature Search Strategy

To provide a comprehensive overview of the current evidence, this narrative review identified relevant literature on massage therapy for NP through a structured search of major scientific databases, including PubMed, Web of Science, Scopus, China National Knowledge Infrastructure (CNKI), China Biomedical Literature Database (CBM), China Scientific Journal Database (VIP), and Wan‐fang. The search covered publications from January 2010 to January 2026 with no language restriction.

The search strategy combined keywords related to both the intervention and the disease condition. Core search terms included “massage,” “Anmo,” “massage therapy,” “neuropathic pain,” “nerve injury,” “analgesia,” and “mechanism.” These keywords were applied individually and in combination using Boolean operators (AND/OR) to broaden the search scope. Studies were considered eligible if they investigated the clinical effects or potential mechanisms of massage and related manual interventions in the context of NP. Both clinical studies and experimental research, including animal models and mechanistic investigations, were included to integrate evidence from different research perspectives. Studies not directly related to NP or lacking relevance to massage or manual mechanical interventions were excluded. In addition, the reference lists of relevant review articles and primary studies were manually screened to identify additional pertinent publications. After removing clearly irrelevant records based on titles and abstracts, studies relevant to the clinical application or mechanisms of massage in NP were included in the qualitative synthesis. Approximately 1714 articles were initially identified, and studies most relevant to the clinical effects and mechanisms of massage in NP were ultimately included for narrative synthesis.

## 2. Clinical Efficacy of Massage: Evidence‐Based Findings and Multidimensional Evaluation

Massage is a manual therapy centered on specific manipulative techniques. Its role in NP management is gradually shifting from an experience‐based adjunctive option to an evidence‐driven component of comprehensive interventions. With improvements in clinical trial design, an increasing body of literature indicates that massage has significant efficacy in relieving certain types of NP, with benefits including improved functional impairment, reduced dependence on analgesic medications, and mitigated comorbid emotional disturbances. This section summarizes the clinical efficacy of massage across etiological subtypes of NP and integrates evaluations of safety and adherence.

### 2.1. Clinical Evidence for Massage in NP

Existing clinical evidence suggests that massage may provide analgesic and functional benefits across several NP‐related conditions, although the certainty of evidence varies by etiology and study design [19]. In lumbar disc herniation (LDH)–related radicular symptoms, randomized clinical studies of massage‐based interventions have reported improvements in pain and disability‐related outcomes [20]. In DPN, a recent meta‐analysis of randomized controlled trials (RCTs) reported reductions in symptom scores and improvements in nerve conduction velocity indices in certain nerves, supporting potential neurophysiological benefits beyond subjective pain reduction [21]. In poststroke populations, a multicenter RCT demonstrated that massage, when integrated with conventional rehabilitation, significantly alleviated upper limb spasticity and promoted motor functional recovery in patients treated during the early poststroke stage. Importantly, these improvements were sustained at medium‐ and long‐term follow‐up, highlighting that massage may contribute to neurofunctional restoration rather than merely transient symptomatic relief [22], although the overall evidence base remains limited.

To further clarify the effectiveness and study characteristics of massage from an evidence‐based perspective, previous clinical trials were synthesized according to the Participants, Intervention, Comparison, Outcome, and Study Design (PICOS) framework. Participants mainly comprised patients with various forms of NP; interventions were primarily standardized massage techniques such as pressing, kneading, pushing, and grasping; comparators were commonly analgesic medications, physical therapy, or sham touch; outcomes focused on pain relief, sensory function, and improvements in quality of life; and study designs were predominantly RCTs. On this basis, a PICOS scale for massage in NP was constructed (see Table 1) to provide a structured framework for subsequent efficacy assessment [20, 22–28].

**TABLE 1:** PICOS summary of clinical research on massage for NP.

	Participants (P)			Intervention (I)		Comparison (C)		Outcomes (O)	Study design (S)
Ge et al. [23]	Adults (≥ 18 years)	HIV‐related peripheral neuropathy	HIV diagnosis; PN symptoms in lower extremities; not taking PN medications; no massage in the previous 7 months	Therapeutic Chinese massage on lower extremities (acupoint pressing, rolling, and kneading manipulations)	25 min/session, 3 sessions/week	Placebo massage (gentle light touch without acupoint stimulation)	25 min/session, 3 sessions/week	PN pain (numeric pain rating); lower extremity function (LEFS); quality of life (SF‐36)	Pilot single‐center double‐blind RCT (*n* = 20)
Cao et al. [20]	Adults (18–55 years)	Lumbar disc herniation (LDH)	Chronic low back pain ≥ 3 months; VAS > 3	Traditional massage (rolling, kneading, acupoint pressing, oblique manipulation)	3 sessions/week for 4 weeks	Lumbar traction	20 min/session, 3 sessions/week for 4 weeks	Clinical efficacy; ODI; lumbar range of motion	Single‐center RCT (*n* = 66)
Sarısoy et al. [22]	Adults (> 18 years)	Non‐Hodgkin’s lymphoma with chemotherapy‐induced peripheral neuropathy	Receiving vincristine chemotherapy; diagnosed neuropathy by electromyography; VAS ≥ 1 neuropathic pain	Foot massage	20 min/session (10 min each foot), 3 sessions/week for 4 weeks	Routine care (no massage)	Same duration (4 weeks)	Neuropathic pain (DN4); pain intensity (VAS)	Randomized controlled trial (*n* = 40)
Cui et al. [24]	Adults (18–65 years)	Cervical radiculopathy	Neck pain ≥ 2 weeks; VAS ≥ 30 mm	Shi’s cervical massage (muscle relaxation, joint adjustment, acupoint pressing)	6 sessions over 2 weeks	Mechanical cervical traction	20 min/session, 6 sessions over 2 weeks	NDI; VAS pain score	Multicenter RCT (*n* = 359)
Gündüz‐Oruç et al. [25]	Adults (18–65 years)	Type 2 diabetic peripheral neuropathy	Diabetes duration ≥ 1 year; no bleeding disorder or skin ulcer	Acupoint massage (LI4, LI11, etc.)	4 kg pressure, 1.5 min/point; 20 min/session for 6 days	No additional intervention	Routine antidiabetic medication only	PQAS pain score; PSQI sleep quality score	Single‐center RCT (*n* = 86)
Lovas et al. [26]	Adults (18–79 years)	Chronic pain after spinal cord injury	≥ 12 months with chronic pain/fatigue	Swedish massage (upper body, light–moderate pressure)	30 min/session, once weekly for 5 weeks	Guided imagery relaxation	Same frequency for 5 weeks	SF‐MPQ pain score; Chalder fatigue score	Single‐center RCT (*n* = 40)
Apichartvorakit et al. [27]	Adults (≥ 18 years)	Peripheral neuropathic pain	DN4 ≥ 4; NRS ≥ 4; stable treatment for the previous 3 months	Court‐type Thai traditional massage (CTTM) plus hot herbal compression; standard drug treatment	Massage 45 min/session + hot herbal compression 15 min/session, 2 sessions/week for 4 weeks	Hot herbal compression	Standard drug treatment + HHC, 2 sessions/week for 4 weeks	NRS pain intensity; NPSI‐T; BPI‐T; EQ‐5D‐5L	Single‐blind RCT (*n* = 28)
Izgu et al. [28]	Adults (≥ 18 years)	Breast cancer with chemotherapy‐induced peripheral neuropathy	Receiving adjuvant paclitaxel; no documented history of CIPN; no neuropathy‐related comorbidities	Classical massage	30 min/session, once weekly for 12 weeks	Usual care (no massage)	Same chemotherapy schedule; no additional intervention	S‐LANSS; EORTC QLQ‐CIPN20; NCS findings	Assessor‐blinded RCT (*n* = 40)

Despite these encouraging findings, the overall strength of clinical evidence supporting massage for NP remains variable. A substantial degree of clinical heterogeneity should be considered when interpreting the results. The included studies involved markedly diverse NP conditions, including HIV‐related peripheral neuropathy, cervical radiculopathy, LDH‐related radicular pain, chemotherapy‐induced peripheral neuropathy, diabetic polyneuropathy, spinal cord injury–related chronic pain, and poststroke spasticity‐related symptoms. These conditions differ considerably in their underlying pathophysiology, which may influence responsiveness to massage. In addition, the intervention protocols varied substantially across studies, including different massage modalities (e.g., Tuina, Swedish massage, classical massage, Thai traditional massage, and acupressure), as well as differences in treatment frequency, duration, and stimulation parameters. Comparator conditions were also heterogeneous, ranging from usual care and no intervention to active controls such as traction, guided imagery, placebo massage, and hot herbal compression.

Therefore, although the current evidence suggests that massage may provide clinical benefits in certain NP‐related conditions, the extent to which these effects can be generalized to specific NP syndromes remains uncertain. These findings should be interpreted with caution when applied to individual clinical contexts.

### 2.2. Risk of Bias and Certainty of Evidence

To evaluate the methodological quality of the clinical studies summarized in Table 1, risk of bias was assessed using the Cochrane Risk of Bias tool. The assessment included several domains, such as random sequence generation, allocation concealment, blinding of participants and personnel, incomplete outcome data, and selective reporting.

Overall, although most studies reported randomization procedures, detailed descriptions of allocation concealment and blinding were often lacking. Because massage therapy interventions are difficult to blind, performance bias may be unavoidable in some trials. In addition, several studies had relatively small sample sizes, which may increase the risk of imprecision.

**FIGURE 1:**
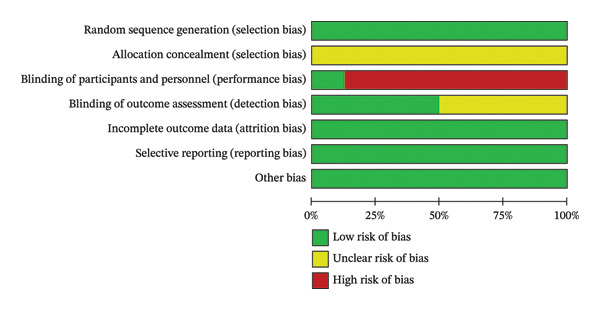
Risk of bias graph of included studies. This schematic overview summarizes the overall risk of bias across included studies, assessed using the Cochrane Risk of Bias tool. The proportions of studies with low, unclear, and high risk are presented for each domain, including random sequence generation, allocation concealment, blinding, incomplete outcome data, and selective reporting.

**FIGURE 2:**
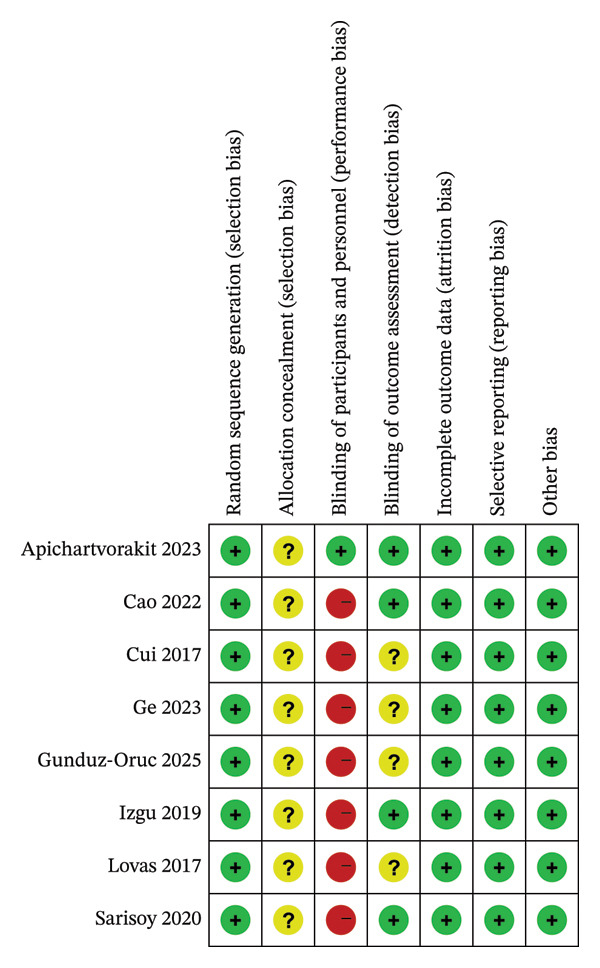
Risk of bias summary of included studies. This schematic overview illustrates the risk of bias assessment for each included study across all domains. Individual studies are evaluated using the Cochrane Risk of Bias tool and categorized as low, unclear, or high risk, allowing visualization of methodological heterogeneity.

**FIGURE 3:**
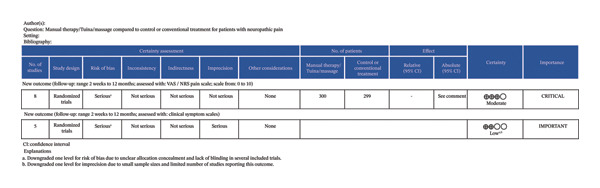
Certainty of evidence assessed by the GRADE framework. This figure summarizes the certainty of evidence for key clinical outcomes of massage/manual therapy in neuropathic pain, evaluated using the GRADE approach. Evidence certainty was rated based on risk of bias, inconsistency, indirectness, and imprecision and categorized as moderate or low.

The certainty of evidence for the main outcomes was further evaluated using the Grading of Recommendations Assessment, Development and Evaluation (GRADE) framework. Overall, the certainty of evidence ranged from low to moderate, mainly due to heterogeneity in intervention protocols, limited sample sizes, and variability in outcome measurements. The detailed results of the risk of bias assessment and evidence certainty grading are presented in Figures 1–3.

These methodological limitations should be explicitly considered when interpreting the reported clinical benefits. In particular, unclear allocation concealment may introduce selection bias, while insufficient blinding of participants and personnel is especially important in massage trials because primary outcomes such as pain intensity and functional status are largely patient‐reported and therefore susceptible to expectation effects and performance bias.

Furthermore, several included studies were pilot trials or had relatively small sample sizes, which may reduce statistical precision and increase the likelihood of overestimating treatment effects. Consistent with the GRADE assessment, which rated the certainty of evidence as low to moderate, these factors suggest that the observed benefits of massage should be regarded as suggestive rather than definitive and require confirmation in larger, well‐designed RCTs.

### 2.3. Effects of Massage on Psychological Status and Quality of Life

NP is frequently accompanied by psychological burdens, including anxiety, depression, sleep disorders, and persistent tension [29]. Pharmacological management often requires concomitant antidepressants or sedatives, increasing the risk of polypharmacy [30]. In contrast, while reducing nociceptive input, massage may promote a more relaxed autonomic state through tactile‐pressure stimulation, thereby gradually relieving negative emotions and psychological tension [31]. Studies indicate that massage can significantly lower anxiety and depression scale scores, reduce attention to pain and catastrophizing, and thus disrupt the vicious cycle of “pain–anxiety–pain exacerbation” [32, 33]. Potential mechanisms may involve reduced serum cortisol levels and increased levels of central neurotransmitters, such as 5‐hydroxytryptamine (5‐HT) and dopamine, which may improve sleep quality [32]. In addition, improvements in sleep quality appear particularly prominent. By reducing nocturnal pain interference, relaxing muscle tension, and shortening sleep onset latency, massage may increase the proportion of deep sleep and enhance overall sleep experience, thereby facilitating recovery of daytime activity. Improved mood and sleep can, in turn, increase tolerance to painful stimuli, producing synergistic benefits at both physical and psychological levels and highlighting the holistic regulatory advantages of massage [32, 34].

### 2.4. Safety and Adherence

In terms of safety, massage has clear advantages over pharmacotherapy [35]. Systematic reviews suggest that serious adverse events during massage are infrequent and are primarily associated with nonprofessional or nonstandard techniques. More commonly, mild and transient effects (e.g., muscle soreness, short‐term pain, or mild discomfort) occur and typically resolve spontaneously after rest [36]. This favorable safety profile makes massage particularly suitable for older adults, individuals with hepatic or renal impairment, and those who are intolerant to analgesic drugs [37]. Moreover, as a contact‐based intervention with a strong humanistic component, massage is generally associated with high patient satisfaction and adherence, which may help build a positive clinician–patient relationship, generate beneficial placebo effects, and further enhance therapeutic outcomes [38, 39].

## 3. Analgesic Effects of Massage via Anti‐Inflammatory Actions

**FIGURE 4:**
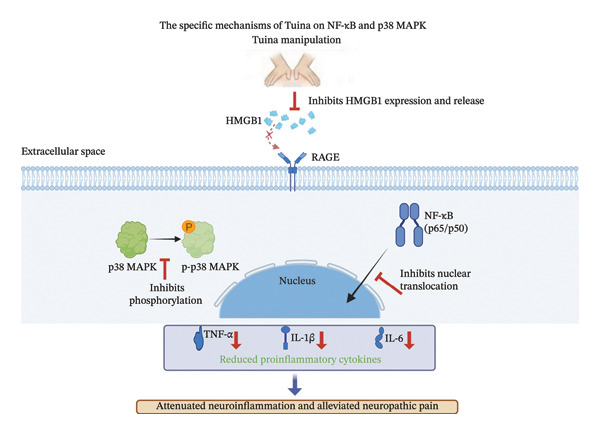
Proposed anti‐inflammatory mechanisms of Tuina in neuropathic pain. This schematic overview summarizes the putative anti‐inflammatory pathways through which Tuina may alleviate neuropathic pain, with a focus on the HMGB1/RAGE axis and its downstream NF‐κB and p38 MAPK signaling pathways. Modulation of these pathways is proposed to reduce the production of proinflammatory cytokines, thereby contributing to the attenuation of neuroinflammation.

Neuroinflammation is an intrinsic defense mechanism that can contribute to tissue repair and the clearance of cellular debris. However, inflammatory stimulation may persist due to tissue injury, infection, autoimmune abnormalities, stress, or drugs, exerting detrimental effects [40]. After peripheral nerve injury, proinflammatory mediators such as bradykinin, histamine, interleukins (ILs), tumor necrosis factor (TNF), and nerve growth factor are released; these factors not only directly trigger inflammatory responses but may also drive central sensitization and thereby induce NP [41]. In this context, studies by Yao et al. and Cao et al. indicate that massage can significantly reduce levels of these inflammatory mediators in spinal nerve ligation animal models and in patient serum, suggesting that massage may exert anti‐inflammatory analgesic effects by regulating IL‐6, TNF‐α, and IL‐1β [20, 42]. Despite these advances, the specific mechanisms by which massage influences inflammatory pathways in NP remain incompletely understood. However, recent experimental evidence suggests that massage may modulate neuroinflammation through several interconnected signaling cascades. Accordingly, Figure 4 provides an integrative overview of the proposed anti‐inflammatory mechanisms, with a focus on the HMGB1/RAGE–NF‐κB and p38 MAPK pathways.

High‐mobility group box 1 (HMGB1) is a prototypical proinflammatory damage‐associated molecular pattern (DAMP) that can be actively secreted by activated macrophages and monocytes or passively released into the extracellular milieu from necrotic cells [43]. Once released, extracellular HMGB1 interacts with pattern recognition receptors, particularly the receptor for advanced glycation end products (RAGE), thereby initiating two major parallel inflammatory signaling pathways: the nuclear factor‐kappa B (NF‐κB) and mitogen‐activated protein kinase (MAPK) pathways. In the latter, p38 MAPK acts as a key downstream effector; activation of these pathways forms an amplification loop that sustains and aggravates inflammation [44, 45]. In NP, HMGB1 is released into the extracellular space. It binds to RAGE expressed on dorsal root ganglion (DRG) neurons and spinal glial cells, which increases the production of proinflammatory cytokines, including IL‐1β and IL‐6. Mechanistically, NF‐κB mainly mediates transcriptional upregulation of inflammatory genes such as IL‐1β and TNF‐α, whereas p38 MAPK is closely associated with cytokine release, glial activation, and central sensitization; together, these processes promote the progression of NP [43, 46]. Notably, HMGB1 can form functional complexes with IL‐1β, activating interleukin‐1 receptor type I (IL‐1R1) and further amplifying inflammatory signaling to exacerbate NP [43]. Consistent with this mechanism, inhibition of HMGB1 signaling has been shown to significantly reduce IL‐1β, IL‐6, and TNF‐α levels, thereby alleviating neuroinflammation [47]. In rat models of compressive nerve injury, Lu et al. (2025) and Tan et al. (2024) reported marked increases in serum IL‐1β, IL‐6, and TNF‐α, along with increased HMGB1 protein and mRNA expression in the spinal dorsal horn. Notably, after 14 days of massage, the massage group showed significantly lower serum levels of IL‐1β, IL‐6, and TNF‐α, as well as reduced HMGB1 protein and mRNA expression, compared with the model group. These findings suggest that massage may effectively attenuate inflammatory responses by targeting HMGB1‐related pathways in the spinal dorsal horn [48].

NF‐κB is a pivotal inflammatory transcription factor that acts synergistically with p38 MAPK and plays a key role in the initiation and maintenance of inflammation and pain signaling. NF‐κB is highly expressed in neurons and microglia in the spinal dorsal horn and the DRG, underscoring its central role in neuroinflammatory regulation [41, 46]. Evidence indicates that NF‐κB and downstream inflammatory mediators, such as IL‐1β and TNF‐α, primarily initiate inflammatory responses and transmit nociceptive signaling in nerve injury models via inflammatory mechanisms [49]. Specifically, HMGB1 release or Toll‐like receptor 4 (TLR4) activation triggered by nerve injury can activate the IκB kinase (IKK) complex (IKKα/IKKβ/IKKγ), promote phosphorylation and degradation of the inhibitor IκBα, and release NF‐κB dimers (p65/p50). These dimers then translocate into the nucleus, bind promoter regions of proinflammatory genes such as IL‐1β and TNF‐α, and enhance transcription and protein synthesis, thereby contributing to a chronic perineural inflammatory microenvironment and serving as a key initiating factor for NP [43, 50]. Interestingly, proinflammatory cytokines released after NF‐κB activation (e.g., IL‐1β and TNF‐α) can in turn bind to their receptors (IL‐1R and TNFR1), trigger intracellular signaling, and further activate the NF‐κB pathway, forming a positive feedback loop of “cytokines ⟶ NF‐κB activation ⟶ more cytokines ⟶ further NF‐κB activation” [51]. In experimental studies using rat models of compressive nerve injury, Sa et al. and Huang et al. demonstrated that massage significantly reduced NF‐κB activation and p65 nuclear translocation [52, 53]. Further, Yao et al. reported that massage not only decreased NF‐κB activation and p65 nuclear translocation but also reduced the expression of the inflammatory cytokines TNF‐α, IL‐1β, and IL‐6 [42]. These findings suggest that targeting the NF‐κB signaling pathway may represent an essential strategy for massage to alleviate NP, providing key theoretical support for its anti‐inflammatory analgesic effects.

The p38 mitogen‐activated protein kinase (p38 MAPK) pathway plays a crucial role in mediating inflammatory responses and pain signal transmission, particularly in neuroglia–neuron interactions. The expression and activation of p38 MAPK in the spinal dorsal horn have been extensively documented, highlighting its importance in neuroinflammatory signaling [54]. Activation of p38 MAPK and its downstream inflammatory mediators (e.g., TNF‐α and IL‐6) has been implicated in nociceptive signaling in both neuropathic and inflammatory pain models [44, 54]. Once activated, p38 MAPK promotes phosphorylation of transcription factors, including activating transcription factor 2 (ATF‐2) and cAMP response element‐binding protein (CREB), thereby enhancing transcription of proinflammatory genes such as TNF‐α and IL‐1β. This process disrupts inhibitory synaptic transmission and facilitates central sensitization, a hallmark of NP [44, 54]. Notably, TNF receptors and interleukin‐1 receptors can initiate intracellular signaling cascades upon ligand binding (TNF‐α and IL‐1β), ultimately activating p38 MAPK, thereby forming a positive feedback loop [51, 55]. Experimental studies in rat models of compressive nerve injury by Yao et al. showed that massage significantly reduced p38 MAPK, phosphorylated p38 MAPK (p‐p38 MAPK), TNF‐α, and IL‐1β levels [56, 57]. Collectively, these data indicate that targeting the p38 MAPK signaling pathway may be an effective strategy for massage to alleviate NP and provide a theoretical basis for its use as an intervention for inflammation‐mediated pain.

## 4. Analgesic Effects of Massage via Regulation of Ion Channels

**FIGURE 5:**
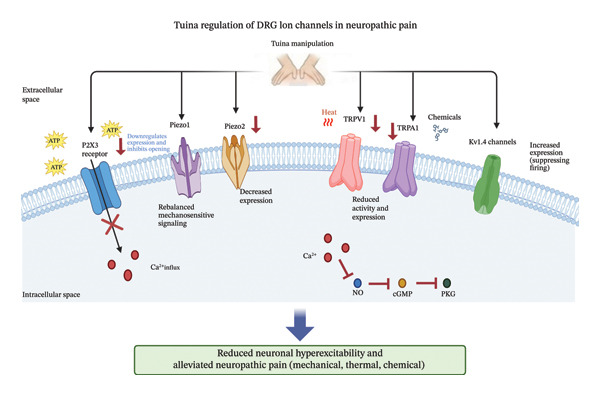
Regulation of dorsal root ganglion (DRG) ion channels by Tuina in neuropathic pain. This schematic overview illustrates the proposed mechanisms by which Tuina may modulate multiple classes of ion channels in dorsal root ganglion neurons, including ligand‐gated, mechanosensitive, transient receptor potential, and voltage‐gated channels. Coordinated regulation of these channels is proposed to rebalance mechanosensitive signaling, reduce aberrant calcium influx and excitatory neurotransmitter release, and thereby attenuate neuronal hyperexcitability associated with mechanical, thermal, and chemical hypersensitivity.

Ion channel dysfunction also contributes to the development of hyperalgesia after peripheral nerve injury. It is widely recognized that peripheral nerve injury disrupts the expression and function of ion channels, leading to neuronal hyperexcitability and, ultimately, hyperalgesia and tactile dysfunction [58]. A key mechanism underlying increased neuronal excitability is the postinjury imbalance or dysfunction of multiple ion channels, which aggravates nociceptive transmission through excessive ion influx and aberrant signal transduction [59]. As an effective intervention, massage has been shown to restore normal neuronal excitability and produce analgesia by modulating the expression and function of ion channels across multiple targets [60, 61]. Although regulatory effects have been reported, the specific mechanisms that target different types of ion channels remain to be systematically elucidated. Figure 5 schematically illustrates the proposed mechanisms by which massage modulates multiple classes of ion channels in DRG neurons, thereby regulating neuronal excitability. The main regulatory pathways are summarized below:

Ligand‐gated ion channels, particularly the P2X3 receptor, are key mediators of nociceptive signal transmission. As an ATP‐gated cation channel, the P2X3 receptor is markedly upregulated after nerve injury and enhances nociceptive transmission by promoting calcium influx and activating neurons [62, 63]. Evidence suggests that massage can downregulate P2X3 receptor expression in the DRG and inhibit channel opening, thereby reducing ATP‐mediated calcium influx, lowering neuronal excitability, and alleviating mechanical allodynia [64]. In animal studies, massage reduced P2X3 receptor expression in the DRG of rats with chronic constriction injury (CCI) by more than 40%, significantly decreased the amplitude of inward currents, and markedly increased mechanical withdrawal thresholds, supporting the effectiveness of this regulatory pathway [65, 66].

Piezo mechanosensitive channels are involved in maladaptive remodeling of mechanical signals after peripheral nerve injury and contribute to mechanical pain abnormalities and tactile dysfunction. Although both Piezo1 and Piezo2 participate in injury‐related mechanical signaling, their expression and functions are highly context‐dependent, with no uniform up‐ or downregulation pattern [67, 68]. Mechanical pain abnormalities appear to result primarily from an imbalance in the allocation of Piezo‐mediated signals between nociceptive and tactile pathways rather than from absolute channel expression levels. Targeting this imbalance may alleviate mechanical hypersensitivity and improve tactile function [69, 70]. In a CCI rat model, Song et al. (2018) observed increased Piezo2 and decreased Piezo1 expression in dorsal root ganglia after injury; following massage intervention, Piezo2 decreased, and Piezo1 increased, paralleling reduced mechanical hypersensitivity [71]. These findings suggest that massage relieves pain by remodeling Piezo‐mediated mechanical responses rather than by simply regulating channel expression.

Enhanced activity of transient receptor potential (TRP) channels, including TRPV1 and TRPA1, constitutes a key pathological basis for heat‐, cold‐, and chemical‐induced pain in peripheral neuropathic pain (pNP). TRPV1 primarily mediates heat nociception and can also respond to chemical stimuli to participate in pain transmission, whereas TRPA1 mainly mediates cold nociception and chemical pain and may also contribute to heat‐related signaling under inflammatory conditions or in nonmammalian species [70, 72]. Following nerve injury, both channels exhibit markedly increased activity, amplifying nociceptive transmission and promoting persistent pain [71, 73]. TRPV1/TRPA1‐mediated calcium influx can activate the NO/cGMP/PKG signaling pathway and induce the release of nociceptive neuroactive substances, thereby further enhancing neuronal excitability [74]. Evidence suggests that massage may reduce sensitivity to thermal, cold, and chemical stimuli by inhibiting nitric oxide (NO) production and modulating downstream cyclic guanosine monophosphate (cGMP) and protein kinase G (PKG) signaling. Within this proposed framework, PKG‐dependent modulation of TRPV1/TRPA1 has been suggested to contribute to the regulation of abnormal calcium influx. Yang et al. (2024) demonstrated that, in rats with minor chronic constriction injury (minor CCI), massage significantly reduced aberrantly elevated TRPV1 and TRPA1 expression in the DRG, downregulated pathway‐related molecules including NO, cGMP, and PKG1, and markedly improved thermal withdrawal latency and mechanical withdrawal thresholds, supporting an essential mediating role of this pathway in massage analgesia [75].

Dysfunction of voltage‐gated sodium, potassium, and calcium channels is a key contributor to neuronal hyperexcitability after peripheral nerve injury [76]. Enhanced NaV1.7/NaV1.8 activity promotes abnormal action potential firing [73], impaired Kv7 channel function increases membrane excitability [77], and overactivation of CaV2.2 augments pain‐related neurotransmitter release, together facilitating nociceptive signal transmission [78]. A‐type voltage‐gated potassium channels primarily regulate firing dynamics rather than membrane stability. Cheng et al. (2025) showed that massage intervention in a CCI rat model increased Kv1.4, Kv3.4, and Kv4.3 expression in IB4‐positive nociceptors while reducing A‐type potassium currents, thereby suppressing neuronal hyperexcitability and alleviating mechanical and thermal hypersensitivity [79]. In addition, in CCI rats treated with high‐voltage long‐duration pulsed radiofrequency (HL‐PRF), a physical modality with specific stimulus features comparable to massage mechanical stimulation, Cai et al. (2022) observed a significant reduction in CaV2.2. Similarly, in CCI rats treated with spinal stimulation (SNS), Liu et al. (2025) reported a substantial decrease in CaV2.2. In both studies, inhibition of CaV2.2 channel function was associated with modulation of neurotransmitter release, including glutamate, substance P, and calcitonin gene‐related peptide (CGRP), thereby contributing to analgesia [80, 81]. Although direct experimental evidence that massage produces analgesia by regulating NaV channels remains lacking, acupuncture and related physical therapies within TCM provide direct support. For example, Huang et al. (2013) and Luo et al. (2022) used low‐frequency electroacupuncture at Zusanli (ST36) and/or Huantiao (GB30) in rodent models of inflammatory pain. They found that, compared with model groups, NaV1.7 expression in the DRG was markedly downregulated (with concurrent downregulation of NaV1.8 in the DRG), thereby alleviating pain sensitization and related behaviors. Further multisample, multicenter studies are needed to clarify the regulatory effects of massage on voltage‐gated ion channels and the underlying mechanisms [82, 83].

In summary, massage may alleviate hyperalgesia and tactile dysfunction after peripheral nerve injury by targeting four significant ion categories: ligand‐gated, mechanosensitive, TRP, and voltage‐gated channels and restoring ion channel homeostasis at both the expression and functional levels, thereby normalizing neuronal excitability. While parts of these regulatory mechanisms have been supported by experimental evidence, the cooperative regulatory networks across different ion channels and the crosstalk among downstream signaling pathways remain to be explored in greater depth.

## 5. Analgesic Effects of Massage via Modulation of Spinal Synaptic Plasticity

**FIGURE 6:**
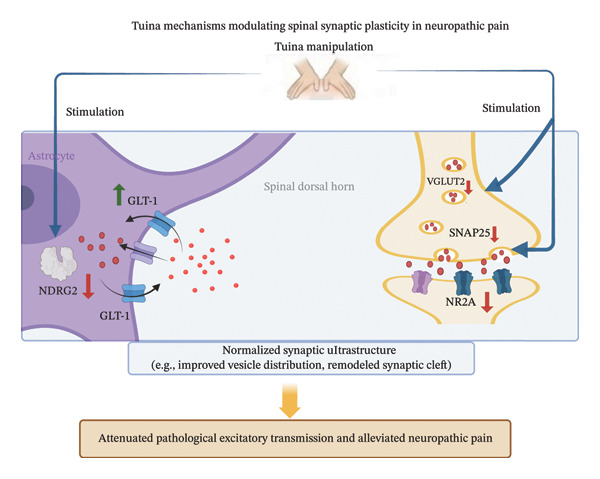
Modulation of spinal synaptic plasticity by Tuina in neuropathic pain. This schematic overview illustrates the proposed mechanisms by which Tuina may regulate synaptic plasticity in the spinal dorsal horn. Tuina is suggested to modulate astrocyte–neuron interactions, particularly through the NDRG2/GLT‐1 pathway, thereby enhancing glutamate clearance and normalizing synaptic ultrastructure. Concurrent regulation of presynaptic and postsynaptic components is proposed to attenuate pathological excitatory transmission, ultimately contributing to the alleviation of neuropathic pain.

In the nervous system, synapses constitute the functional structural basis for neuronal connectivity. Their morphological and functional plasticity support neural signal transmission and intercellular communication [84]. Evidence indicates that synaptic plasticity in the dorsal horn of the spinal cord drives nociceptive transmission and is a critical mechanism for the development and maintenance of NP [85]. Dysregulated glutamatergic transmission and glia–synapse interactions can amplify excitatory signaling and contribute to central sensitization. Marked alleviation of NP has been closely linked to the astrocytic N‐myc downstream‐regulated gene 2 (NDRG2), which regulates synaptic plasticity by modulating glutamate clearance via the glutamate transporter 1 (GLT‐1), reflecting bidirectional glia–synapse interactions [86, 87]. Based on these findings, Figure 6 illustrates the proposed mechanisms by which massage modulates synaptic plasticity in the dorsal horn of the spinal cord.

In a rat model of sciatic nerve CCI, Zhang et al. (2024) found that massage (pressing and kneading at the Weizhong acupoint) regulated the astrocytic NDRG2/GLT‐1 pathway, including downregulation of NDRG2 and upregulation of GLT‐1 in the spinal dorsal horn, reduction of glutamate concentration in the synaptic cleft, and widening of the synaptic cleft. These findings indicate that massage can reverse CCI‐induced ultrastructural synaptic abnormalities and modulate spinal synaptic structural and functional plasticity, thereby producing analgesia [88]. Jiang et al. further showed that massage reduced the expression of synapse‐associated protein 25 (SNAP25), vesicular glutamate transporter 2 (VGLUT2), and N‐methyl‐D‐aspartate receptor 2A (NR2A) in the spinal dorsal horn, effectively decreasing the level of the pain‐related neurotransmitter glutamate and improving synaptic ultrastructure of dorsal horn neurons (more uniform synaptic vesicle distribution, reduced postsynaptic density area, and a narrowed synaptic cleft). This regulatory mechanism may modulate synaptic plasticity and suppress synaptic hyperactivity, thereby exerting analgesic effects on LDH‐associated NP [89]. Although specific ultrastructural features (e.g., changes in synaptic cleft width) may vary across disease models, stages, and measurement targets, both studies consistently support the notion that massage attenuates pathological excitatory transmission and normalizes dorsal horn synaptic remodeling. Collectively, these findings elucidate molecular mechanisms by which massage regulates spinal synaptic plasticity and provide a theoretical basis for its use as an intervention for NP.

## 6. Analgesic Effects of Massage via Modulation of Brain Function

The brain is the final site of pain generation and plays a central role in the transformation, transmission, and modulation of neural signals [90]. NP can be regarded as the consequence of disrupted coordination among brain networks involved in pain perception, emotional regulation, and cognitive appraisal [91, 92]. In chronic pain states, abnormal structural plasticity in specific brain regions interacts with functional connectivity imbalances across whole‐brain networks, thereby promoting pain chronification [93, 94]. The central analgesic effects of massage may be understood along two complementary dimensions: (1) structural regulation, which targets synapses, signaling pathways, and neuronal microarchitecture within core pain‐related regions and (2) network‐level regulation, which reshapes coordinated function across distributed brain networks. Together, these dimensions form a “local targeting–global integration” regulatory pattern, providing a neurobiological framework for understanding the central effects of massage in chronic pain.

### 6.1. Structural Regulation: Local Targeting of Core Pain‐Related Brain Regions

Central pain coding depends on the coordinated activity of key brain regions, including the anterior cingulate cortex (ACC), the primary and secondary somatosensory cortices (S1/S2), and the insular cortex (IC) [95]. In NP, these regions frequently exhibit maladaptive functional reorganization (altered activity and connectivity), a central feature of the disorder [96]. Massage may attenuate central amplification of pain signals by modulating local structure and function within these regions.

As an affective–sensory integrative hub for pain, the ACC contributes not only to encoding pain intensity but, more critically, to mediating pain‐related negative emotions such as anxiety and aversion. Abnormal synaptic plasticity in the ACC (e.g., excessive activation of N‐methyl‐D‐aspartate (NMDA) receptors and enhanced synaptic transmission) provides a fundamental basis for central sensitization in chronic pain [97, 98]. In CCI rats, Lian et al. (2025) demonstrated that massage downregulated the overactivated calcium/calmodulin‐dependent protein kinase II (CaMKII) signaling pathway in the ACC, inhibited aberrant expression of postsynaptic density protein 95 (PSD‐95), and reversed pathological ultrastructural remodeling of ACC synapses, thereby reducing pain‐related anxiety‐like behaviors. This mechanism may form a central–peripheral synergistic analgesic network, together with massage regulation of spinal synaptic plasticity [3, 99]. Clinical evidence further supports this possibility. In patients with knee osteoarthritis pain, although changes in ACC blood oxygen level‐dependent (BOLD) signals were not explicitly concluded, the observed correlation between reductions in ACC BOLD activity and pain relief suggests that modulation of ACC reactivity may represent a potential central substrate of massage analgesia, thereby providing a rationale for subsequent multimodal neuroimaging investigations of massage‐related central mechanisms [100].

The primary and secondary somatosensory cortices (S1/S2) are core regions for spatial localization and pain discrimination. S1 (primary somatosensory cortex) primarily supports spatial localization, intensity encoding, and basic modality discrimination of sensory stimuli [101], whereas S2 (secondary somatosensory cortex) further integrates information from S1 and other areas, participating in more complex sensory discrimination, object recognition, and linking sensory information with emotion and memory [102]. After chronic nerve injury, cortical representation areas may undergo reorganization, leading to hyperalgesia and abnormal touch perception. In a clinical experiment, Xing et al. (2021) found that the amplitude of low‐frequency fluctuation (ALFF) in the contralateral somatosensory cortex of the affected hindlimb was lower in the model group than in the control group, indicating reduced spontaneous activity following peripheral nerve injury (PNI). Moreover, after 4 weeks of massage intervention, compared with the model group and the sham massage group, the massage group exhibited significantly higher ALFF in the contralateral somatosensory cortex, suggesting that massage therapy may restore neuronal activity [103]. In animal experiments, Wu et al. showed that massage increased gamma‐aminobutyric acid (GABA) expression in GABAergic neurons in S1 and inhibited the release of excitatory glutamatergic neurotransmitters. By rebalancing inhibitory and excitatory neural circuits, massage reduced excessive sensitization of the somatosensory cortex [104].

The IC, an interoceptive hub, participates in sustained pain perception and affective projection and serves as an important region that integrates pain sensation with emotion, reward, cognition, and memory [105]. Evidence from neuroimaging studies of massage‐related acupoint stimulation suggests that IC‐centered circuits may be engaged during clinical symptom improvement. In a longitudinal pilot fMRI study, auricular point acupressure was accompanied by clinically meaningful reductions in neuropathic symptoms and concurrent alterations in intrinsic brain networks, including changes at key nodes of the salience network, including the insula and ACC [106]. Although direct evidence for massage modulation of the IC is still accumulating, mechanistic findings from related external therapies in TCM (e.g., acupuncture) provide a supportive context. In animal studies, Xiao et al. (2023) reported that electroacupuncture in mouse pain models may block pain‐related aversive memory behaviors by activating GABAergic neurons and inhibiting kappa opioid receptors (KORs) in the IC [107]. In a clinical study of central pain, Liang et al. compared two acupuncture approaches. They found that electroacupuncture at Jing points, combined with stimulation at Jieju points, produced greater analgesia than stimulation at Jing points alone. More strongly suppressed activity in pain‐processing brain regions, both needling protocols elicited significant inhibitory activity in the inferior parietal lobule (IPL), ACC, and IC, thereby producing analgesic effects. These findings suggest that modulation of IC‐related pain processing may represent a plausible central target for massage and warrant further direct verification [108].

### 6.2. Network (Grid) Regulation: Global Coordinated Remodeling of Brain Networks

Brain function relies on network‐level coordination across multiple regions. Chronic pain disrupts the balance of functional connectivity within these networks. The holistic advantage of massage is reflected in its grid‐like remodeling of brain networks, particularly the default mode network (DMN) and the emotion–pain integration network.

The DMN is a core network that, during rest, supports self‐referential processing, emotion regulation, and memory integration. Key nodes include the medial prefrontal cortex (mPFC), posterior cingulate cortex (PCC), precuneus, and thalamus [109]. Chronic NP can disrupt DMN functional connectivity, impair the DMN “activation–inhibition balance,” and lead to abnormal regulation involving the mPFC, PCC, and amygdala, thereby exacerbating pain perception and cognitive/emotional disturbances [110]. Using resting‐state functional magnetic resonance imaging (fMRI), Chen et al. demonstrated widespread DMN connectivity abnormalities in patients with LDH‐related NP, including reduced regional homogeneity (ReHo) and abnormal variance of dynamic functional connectivity (dFC), indicating dysfunction of DMN core nodes (e.g., the mPFC and precuneus). Compared with healthy controls (HCs), patients with LDH showed significantly lower ReHo in the left orbital middle frontal gyrus and increased dFC variance in the left precuneus. The left orbital middle frontal gyrus is in the mPFC, a core hub of the DMN that supports emotion regulation and internal attention allocation [18, 111, 112]. Abnormal dFC variance suggests impaired dynamic coordination within the DMN, which may contribute to persistent pain and emotional comorbidities in LDH patients [113, 114]. After 4 weeks of massage, functional connectivity within the DMN tended to normalize, and this remodeling was positively correlated with pain relief and improvements in quality of life, suggesting that massage may partially reverse chronic pain‐related network remodeling [18]. In animal studies, Wu et al. (2023) found markedly reduced neuronal activity in the mPFC of CCI rats; massage upregulated brain‐derived neurotrophic factor (BDNF) expression in the mPFC, promoted neuronal survival and synaptic repair, and restored normal activity levels in DMN core nodes [107].

Abnormal interactions between the DMN and the pain matrix (ACC and IC) constitute an essential pathological link in chronic pain [115]. Accordingly, it is plausible that massage may influence inter‐network coupling between the DMN and pain‐processing regions, thereby reducing pain‐related interference with internally directed cognition and contributing to improvements in pain‐associated cognitive and affective disturbances. This network‐level balancing effect may represent one mechanism by which massage complements interventions that primarily target single molecular pathways.

### 6.3. Regulation of the Emotion–Pain Integration Network

NP and emotional disorders such as anxiety and depression often co‐occur. Dysfunction of the emotion–pain integration network, comprising the hippocampus, amygdala, hypothalamus, and related regions, is a key contributor to pain chronification and emotional comorbidity [116, 117]. Massage not only alleviates pain symptoms but may also modulate this network, reducing emotion‐related amplification of pain [118, 119].

The hippocampus is a core region for learning, memory, and emotion regulation; hippocampal neuronal injury and reduced synaptic plasticity can facilitate maintenance of chronic pain [120]. In CCI rats, Wang et al. (2024) found that massage upregulated mRNA and protein expression of sirtuin 1 (SIRT1), BDNF, and its high‐affinity receptor, tropomyosin receptor kinase B (TrkB), in the hippocampus, thereby activating the SIRT1/BDNF/TrkB signaling pathway. Activation of this pathway markedly improved structural abnormalities of CA1 neurons, increased the number of Nissl‐positive cells, repaired damaged dendritic spines, and enhanced the structural plasticity of hippocampal neurons. In addition, this mechanism may improve abnormal pain‐related memory consolidation and, by modulating hippocampus–ACC functional connectivity, alleviate pain‐induced anxiety‐like behaviors [121]. A similar link between hippocampus–ACC connectivity modulation and anxiety‐like behavior has also been reported in related work [101].

**FIGURE 7:**
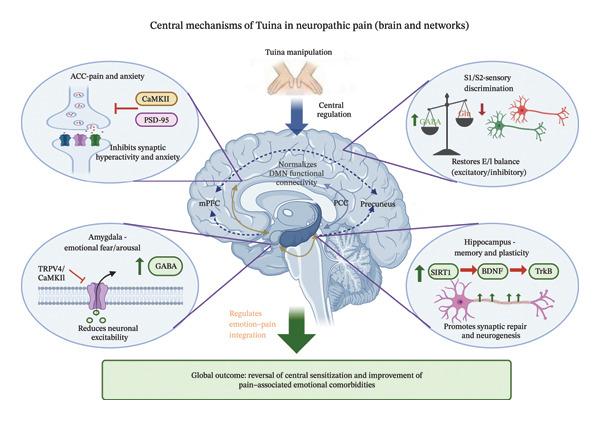
Central mechanisms of Tuina in neuropathic pain at the brain and network levels. This schematic overview illustrates the proposed central mechanisms by which Tuina may modulate neuropathic pain through coordinated regulation of key pain‐related brain regions and large‐scale functional networks. Tuina is suggested to normalize default mode network (DMN) functional connectivity and to modulate activity within regions involved in sensory processing, emotion–pain integration, memory, and affective regulation, including the anterior cingulate cortex, primary/secondary somatosensory cortex, amygdala, and hippocampus. These central effects are proposed to contribute to the reversal of central sensitization and the improvement of pain‐associated emotional comorbidities.

As an emotional center, the amygdala can amplify the negative affective experience of pain when excessively activated, forming a vicious cycle of “pain–anxiety” [122, 123]. Gulianna et al. reported that massage reduced neuronal excitability in the amygdala by inhibiting the overactivation of the TRPV4–CaMKII signaling pathway, thereby decreasing calcium influx and the release of excitatory neurotransmitters. Meanwhile, massage increased synthesis and release of GABA in the amygdala and strengthened inhibitory neurotransmission, thereby disrupting the pathological loop in which emotions amplify pain [102, 104]. In addition, regulation of the hypothalamic–pituitary–adrenal (HPA) axis may also contribute to emotion–pain integration. By attenuating HPA axis overactivation, massage can reduce cortisol release and alleviate stress responses and emotional dysregulation associated with chronic pain [108, 18]. Taken together, these findings indicate that massage exerts central analgesic effects through coordinated regulation of multiple pain‐related brain regions and large‐scale functional networks. Figure 7 provides an integrative schematic overview of the central mechanisms underlying massage‐mediated modulation of NP.

## 7. Conclusion

Massage is a long‐standing traditional therapy that has attracted increasing attention as a nonpharmacological option for relieving NP. Integrating available evidence, massage not only demonstrates favorable analgesic effects in clinical practice but also exhibits a multidimensional, multicomponent regulatory profile in mechanistic studies. Specifically, massage may alleviate NP through coordinated modulation across multiple levels, including anti‐inflammatory regulation, ion channel homeostasis, spinal synaptic plasticity, and pain‐related brain function and network connectivity. These features highlight massage’s potential as a safe and integrative component of multimodal NP management.

Overall, massage, characterized by safety, effectiveness, and regulatory potential at the system level, may offer unique advantages in the treatment of NP. With the integration of modern research methods and advances in standardization, massage may become an increasingly important element in comprehensive NP management, promoting constructive integration between traditional and modern medicine and supporting the development of more precise, mechanism‐informed pain treatment strategies.

## 8. Limitations and Future Perspectives

This article represents a narrative review based on representative literature rather than a systematic review, and the findings should therefore be interpreted as a conceptual synthesis of current evidence. Despite the promising potential of massage in NP, several important limitations should be acknowledged. Across existing studies, substantial heterogeneity exists in massage manipulations, including force or intensity parameters, treatment frequency, course duration, and reporting standards, with no universally accepted framework for intervention description. Such inconsistencies may introduce variability in efficacy estimates and limit comparability across clinical trials.

It should also be noted that not all studies have reported consistent or strongly supportive findings regarding the efficacy or mechanisms of massage. Some clinical trials have reported modest or short‐term improvements in pain outcomes, and in certain cases the observed benefits were comparable to other conservative interventions such as physical therapy or traction. In addition, heterogeneity in treatment protocols, practitioner experience, and outcome measurements across studies may contribute to variability in reported effects.

From a mechanistic perspective, although multiple molecular and neural pathways have been implicated, the current evidence remains incomplete. Much of the available mechanistic evidence derives from animal models or small‐scale experimental studies, and the specific signaling cascades directly targeted by massage have not yet been fully clarified. Moreover, the interactions between peripheral molecular regulation, spinal synaptic plasticity, and large‐scale brain network remodeling remain insufficiently understood. The lack of standardized stimulation parameters—such as force, frequency, duration, and targeted anatomical regions—also limits reproducibility and cross‐study comparability.

Future research should therefore prioritize several directions. First, standardized massage protocols and reporting checklists should be developed to improve reproducibility and facilitate evidence synthesis. Second, rigorously designed multicenter RCTs with adequate sample sizes, longer follow‐up periods, and clinically meaningful endpoints are required to strengthen translational evidence. Third, advanced neuroscience tools—including fMRI, positron emission tomography, and multiomics approaches—should be integrated to identify objective biomarkers, characterize brain network dynamics, and clarify the links between molecular regulation and system‐level remodeling.

Finally, given the complexity and multitarget nature of massage, future studies should avoid attributing its therapeutic effects to a single dominant mechanism. Different NP etiologies and disease stages are likely to involve distinct primary targets and pathways, reflecting massage’s multilevel regulatory actions across peripheral, spinal, and central systems. An integrative research framework that incorporates inflammation, ion channel regulation, spinal synaptic plasticity, and brain network modulation may ultimately support mechanism‐ and phenotype‐specific treatment strategies and optimize clinical implementation.

**Nomenclature**
ACC: Anterior cingulate cortex
ALFF: Amplitude of low‐frequency fluctuations
BDNF: Brain‐derived neurotrophic factor
CaMKII: Ca^2+^/calmodulin‐dependent protein kinase II
CaV: Voltage‐gated calcium channel
CCI: Chronic constriction injury
CGRP: Calcitonin gene‐related peptide
DAMP: Damage‐associated molecular pattern
DMN: Default mode network
DRG: Dorsal root ganglion
E/I balance: Excitatory/inhibitory balance
fMRI: Functional magnetic resonance imaging
GLT‐1: Glutamate transporter 1
Glu: Glutamate
GABA: γ‐aminobutyric acid
HMGB1: High‐mobility group box 1
IL‐1β: Interleukin‐1 beta
IL‐6: Interleukin‐6
Kv: Voltage‐gated potassium channel
mPFC: Medial prefrontal cortex
NaV: Voltage‐gated sodium channel
NDRG2: N‐myc downstream‐regulated gene 2
NF‐κB: Nuclear factor‐kappa B
NP: Neuropathic pain
P2X3: Purinergic receptor P2X3
PCC: Posterior cingulate cortex
PHN: Postherpetic neuralgia
Piezo1/2: Mechanosensitive Piezo ion channels 1 and 2
ReHo: Regional homogeneity
S1/S2: Primary/secondary somatosensory cortex
SNAP25: Synaptosomal‐associated protein 25
SP: Substance P
TCM: Traditional Chinese medicine
TNF‐α: Tumor necrosis factor alpha
TRPA1: Transient receptor potential ankyrin 1
TRPV1: Transient receptor potential vanilloid 1

## Author Contributions

Yiting Guo contributed to conceptualization, literature search, data curation, writing–original draft, figure preparation, and writing–review and editing. Yi Zhong contributed to literature retrieval, literature screening, and writing–review and editing. Xin Yun Chia contributed to data curation, literature screening, writing–review and editing. Xiaoqiu Wang contributed to data curation, formal analysis, and writing–review and editing. Bin Xiao contributed to conceptualization, supervision, critical suggestions, writing–original draft, and writing–review and editing.

## Funding

This work was supported by the National Natural Science Foundation of China (82105041).

## Disclosure

All authors approved the submitted version.

## Ethics Statement

The authors have nothing to report.

## Conflicts of Interest

The authors declare no conflicts of interest.

## Data Availability

Data sharing is not applicable to this article, as no datasets were generated or analyzed during the current study.
